# Long Non-Coding RNAs Differentially Expressed in Swine Fetuses

**DOI:** 10.3390/ani14131897

**Published:** 2024-06-27

**Authors:** Francelly G. Campos, Adriana M. G. Ibelli, Maurício E. Cantão, Haniel C. Oliveira, Jane O. Peixoto, Mônica C. Ledur, Simone E. F. Guimarães

**Affiliations:** 1 Laboratory of Animal Biotecnology, Department of Animal Science, Universidade Federal de Viçosa, Viçosa 36570-000, MG, Brazil; francelly.campos@ufv.br (F.G.C.); hanielcedraz@gmail.com (H.C.O.); 2 Embrapa Suínos e Aves, Concordia 89715-899, SC, Brazil; adriana.ibelli@embrapa.br (A.M.G.I.); mauricio.cantao@embrapa.br (M.E.C.); jane.peixoto@embrapa.br (J.O.P.); monica.ledur@embrapa.br (M.C.L.); 3 Programa de Pós-Graduação em Ciências Veterinárias, Universidade Estadual do Centro Oeste, Guarapuava 85040-167, PR, Brazil; 4 Programa de Pós-Graduação em Zootecnia, Universidade do Estado de Santa Catarina, UDESC-Oeste, Chapecó 89815-630, SC, Brazil

**Keywords:** bioinformatics, chromosome Y, transcriptome

## Abstract

**Simple Summary:**

Long non-coding RNAs (lncRNAs) are a type of RNA that does not produce proteins but plays crucial roles in diverse metabolic processes, including cellular development and differentiation, the regulation of protein production, the inactivation of genes and chromosomes, and gamete formation. Despite their recognized importance, there is a limited understanding of how these molecules function in pigs, particularly during fetal development. For this reason, we conducted this exploratory work, in which we aimed to identify and characterize potential novel lncRNAs in the initial fetal phase of pigs. We have not only identified potentially new lncRNAs but also identified that some of them have sex-specific expression. In the current study, hundreds of potential and already described lncRNAs were identified This allows for the expansion of our knowledge about genomics and transcriptomics in pigs, while also providing novel RNA sequences for the genomic databases of these species. This is particularly important as we are providing data for the international community regarding a research theme that is at its early stages. The potential novel lncRNAs identified in our study provide a basis for future research on their biological functions in a highly important livestock species.

**Abstract:**

Long non-coding RNAs (lncRNAs) are non-coding transcripts involved in various biological processes. The Y chromosome is known for determining the male sex in mammals. LncRNAs on the Y chromosome may play important regulatory roles. However, knowledge about their action mechanisms is still limited, especially during early fetal development. Therefore, we conducted this exploratory study aiming to identify, characterize, and investigate the differential expression of lncRNAs between male and female swine fetuses at 35 days of gestation. RNA-Seq libraries from 10 fetuses were prepared and sequenced using the Illumina platform. After sequencing, a data quality control was performed using Trimmomatic, alignment with HISAT2, and transcript assembly with StringTie. The differentially expressed lncRNAs were identified using the limma package of the R software (4.3.1). A total of 871 potentially novel lncRNAs were identified and characterized. Considering differential expression, eight lncRNAs were upregulated in male fetuses. One was mapped onto SSC12 and seven were located on the Y chromosome; among them, one lncRNA is potentially novel. These lncRNAs are involved in diverse functions, including the regulation of gene expression and the modulation of chromosomal structure. These discoveries enable future studies on lncRNAs in the fetal stage in pigs.

## 1. Introduction

In pigs, the fetal phase is a complex and highly regulated process, which involves a series of molecular and cellular events important for development and growth. Thus, genetic regulation plays fundamental roles in the organization of these processes, and long non-coding RNAs (lncRNAs) emerge as important regulators of gene expression [1].

LncRNAs are transcripts spanning more than 200 nucleotides with reduced ability to encode proteins and low sequence conservation between species, playing essential roles in gene regulation [2,3]. Generally, they are associated with cellular processes that include epigenetic modifications, both transcriptional and post-transcriptional [4,5]. In pigs, lncRNAs have been described to play vital roles in a wide range of biological processes, including the regulation of adipocyte differentiation and lipid metabolism [6], embryonic development [7,8], muscle development [9], and immune regulation [10].

Studies in humans and mice have shown that certain lncRNAs are associated with sex chromosomes and specific functions in sex differentiation, germ cells meiosis, and the expression of sex genes [11,12,13,14]. Furthermore, in these species, conserved lncRNAs have been identified as active participants in the epigenetic regulation of the X chromosome in females [15,16,17]. In contrast, few studies have investigated the biological importance of Y chromosome non-coding RNAs [18].

The Y chromosome lncRNAs are involved in spermatogenesis [19], in the regulation of spermatogonial stem cell proliferation [20,21], germ cell meiosis, and early brain development [22]. Such findings demonstrate the importance of conducting more studies on lncRNAs, especially on the Y chromosome.

Despite the significant advances in understanding lncRNAs and their relationship with sexual dimorphism, many aspects of this interaction still need to be explored in detail to fully understand their impact on early mammalian development, such as in pigs, in which there is still a gap in the identification of the role of lncRNAs in the sex chromosomes’ function and in sex determination. This class of RNAs represents not only a new research target but also an example of how discoveries and growing evidence change existing paradigms and ways of thinking. Therefore, in this study, lncRNAs were identified, and their expression profile between male and female swine fetuses at 35 days of gestation was determined.

## 2. Materials and Methods

### 2.1. Declaration of Ethics

All experimental procedures to generate our results were approved by the Ethics Committee on the Use of Animals of the Universidade Federal de Viçosa (UFV), Minas Gerais, Brazil (protocol no. 06/2017).

### 2.2. Animal Experiment

The present study is part of a previous experiment designed to understand the effects of L-arginine supplementation in the maternal diet [23], the identification of differentially expressed coding genes [24], and the sex determination of the conceptuses [25]. Briefly, at 35 days of gestational age, the gilts were slaughtered and the fetuses (females = 4, males = 6) were quickly washed with a PBS (phosphate-buffered saline) solution, individually identified, stored in liquid nitrogen, and transported to the Animal Biotechnology Laboratory (LABTEC) of the Department of Animal Science at UFV. For this study, ten 35-day-old fetuses from six sows supplemented with 1% L-arginine (ARG) and without supplementation (CONT) were used to identify differentially expressed lncRNAs.

### 2.3. RNA Extraction and Library Preparation

Total RNA extraction from ten 35-day-old fetuses was performed with TRIzol (Invitrogen, San Diego, CA, USA). The fetuses were macerated (100 mg) and mixed with TRIzol (1 mL) by vortexing and then incubated for 5 min at room temperature (RT). Then, 200 µL of chloroform was added, shaken vigorously for 15 s, and incubated at RT for 5 min. Centrifugation was performed at 11,000× *g* at 4 °C for 15 min. Approximately 600 µL of the upper clear aqueous phase containing only RNA was carefully removed and transferred to a new tube, and 600 µL of 70% ethanol was added and mixed by inversion.

After RNA extraction, quantification was performed with a QUBIT fluorimeter (Thermo Scientific, Waltham, MA, USA) and integrity was determined on a 1.0% agarose gel. In addition, the Agilent 2100 BioAnalyzer (Agilent Technologies, Santa Clara, CA, USA) was used for integrity measurement, in which samples with RNA integrity number (RIN) greater than eight were used for library preparation. RNA libraries were prepared using the Illumina Stranded Total RNA Prep Ligation with Ribo-Zero Plus for ribosomal RNA depletion, and the libraries were sequenced with the Illumina HiSeq2000 platform (Illumina, Inc.; San Diego CA, USA).

### 2.4. Quality Control, Mapping, and Transcriptome Assembly

The baqcom pipeline (https://github.com/hanielcedraz/BAQCOM accessed on 22 September 2023) was used for quality control (QC) and mapping. QC was carried out using Trimmomatic v. 0.39 [26], removing short reads (<70 bp), low-quality reads (QPhred < 20), and adapter sequences. The remaining sequences were used for further analysis. Clean reads from each sample were then aligned against the reference genome (Sus scrofa11.1) using the Ensembl 107 annotation (https://www.ensembl.org/Sus_scrofa accessed on 22 September 2023) with HISAT2 [27], a program recommended to be used along with StringTie pipeline.

The mapping results were analyzed using StringTie v2.2.1 [28], which performed the assembly and reconstruction of the transcripts aligned to the reference genome (Sus scrofa 11.1, annotation 107). The parameters defined by default were used, generating a GTF file of transcripts for each sample (Supplementary Table S1). After aligning and assembling the transcripts, filtering was performed using the value of Fragments Per Kilobase Million (FPKM), where only values >0.5 remained in the analysis. Afterward, a merge was performed using a StringTie tool to join in the sample transcripts with the provided reference annotation, resulting in a single GTF file.

### 2.5. Identification of Potentially New lncRNAs

**Figure 1:**
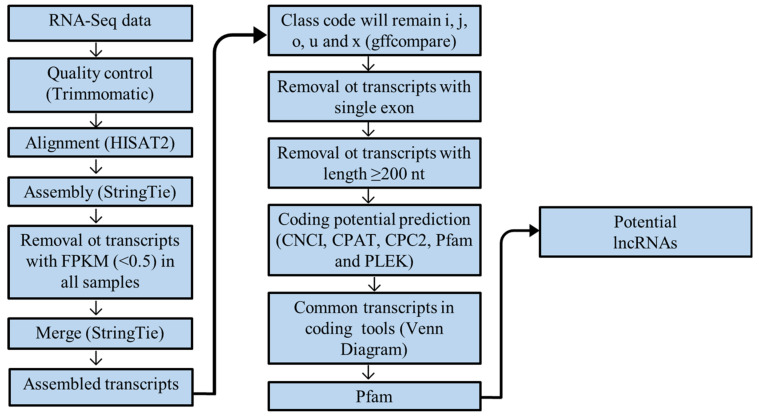
Pipeline used for the identification of potential lncRNAs.

A highly stringent pipeline was performed to identify potential lncRNAs (Figure 1). The gffcompare, a complementary tool of the StringTie package, was used to compare the assembled transcripts to the reference genome aiming to identify transcripts not yet annotated. Of all classes, only non-coding ones, such as “i”, a transfer match entirely within a reference intron (intronic); “j”, at least one splice junction is shared with a reference transcript; “o”, generic exonic overlap with a reference transcript; “u”, unknown, intergenic transcript (intergenic); and “x”, exonic overlap with reference on the opposite strand (antisense), were maintained for downstream analysis.

Then, the single exon transcripts were removed to eliminate unreliable transcripts. Next, the gffread program (https://ccb.jhu.edu/software/stringtie/gff.shtml#gffread accessed on 22 September 2023) was used to generate a fasta file. From this fasta file, new filtering was performed where transcripts ≥200 nt were retained for analysis. Soon after, four coding prediction programs were used: the Coding Potential Calculator (CPC2) [29], Coding-Non-Coding Index (CNCI) [30], Predictor of long non-coding RNAs and messenger RNAs based on an improved k-mer scheme (PLEK) [31], and Coding Potential Assessment Tool (CPAT) [32].

CPC2 evaluates the transcript’s coding potential based on biological sequence characteristics, including homology with known protein sequences and the presence and quality of ORFs [29]. The CNCI classifies protein-coding and non-coding sequences through the independent nucleotide triplet profile and a score lower than zero indicates that it is a non-coding transcript [30]. PLEK is a free alignment tool based on the k-mer frequencies of sequences [31]. CPAT determines the coding and non-coding ability of a transcript by building a logistic regression model based on length, ORF coverage and calculating Fickett and Hexamer scores, and a score lower than 0.364 indicates a non-coding transcript [32].

A Venn diagram was used to identify potential lncRNAs common to all prediction tools, and the transcripts were evaluated using the database of protein families (Pfam). Pfam is a classification system for the annotation of protein domains and is used to identify domains of known protein families. It establishes a statistical model (profile hidden Markov model, HMM) of the amino acid sequence for each family through the alignment of the protein sequence and the mismatched transcript, determining whether or not it is a potential non-coding transcript [33]. Thus, only transcripts that passed through all the filters were classified and considered candidate lncRNAs.

The code for the complete pipeline can be found at: https://ainfo.cnptia.embrapa.br/digital/bitstream/item/262373/1/SDoc-244.pdf accessed on 16 December 2023.

### 2.6. Identification of Differentially Expressed (DE) lncRNAs

For differential gene expression analysis, StringTie was used to calculate transcripts per million (TPM), which was the percentage of FPKM, calculated based on the length of fragments and count of reads mapped to this fragment. The analysis was performed using the known lncRNAs and potentially new assembled lncRNAs in the R language through the limma package [34,35]. Limma was employed to meet the experimental criteria, as we used a nested factorial design in the data analysis. The code used for the analysis is provided in the Supplementary Material (Supplementary File S1). DE lncRNAs were obtained through contrasts between sexes. Positive and negative log2-fold changes (logFC) indicate the upregulation and downregulation of DE lncRNAs, respectively. The lncRNAs presented a *p*-value adjusted for the false discovery rate (FDR) < 0.05, according to the Benjamini–Hochberg method [36].

### 2.7. Target Gene Prediction and Functional Annotations

To annotate the function of the DE lncRNAs, we searched for neighboring genes and considered them as potential targets; it has been reported that lncRNA function depends on protein-coding genes by cis- and trans-action elements [37,38]. The Integrative Genomics Viewer (IGV) [39] was used to visualize the location of the DE lncRNAs and Ensembl was used to identify neighboring genes around 300 kb upstream and downstream of the DE lncRNAs. The functional annotation was performed using the Database for Annotation, Visualization, and Integrated Discovery (DAVID) tool.

## 3. Results

### 3.1. Mapping and Assembly of Transcripts

On average, 31.4 million reads per sample were generated for each fetus, and after data quality control, an average of 29.6 million reads per sample remained. The reads of all samples after quality control were mapped against the porcine reference genome (Sus scrofa11.1 ensembl 107). The percentage of HISAT2 alignment of all samples was between 83 and 85% (Table 1).

### 3.2. Identification and Characterization of Potentially New lncRNAs

**Figure 2:**
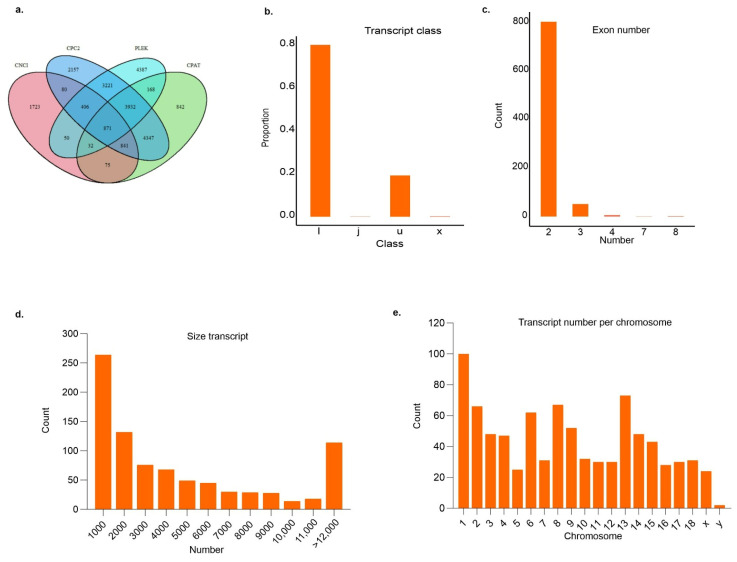
(**a**) Venn diagram of the lncRNAs identified by the CNCI, CPC2, PLEK, and CPAT programs. (**b**) Percentage of lncRNA classes (i—intronic; j—potentially new isoform; u—intergenic; and x—antisense). (**c**) Exon number in the lncRNAs. (**d**) Size (bp) of transcripts. (**e**) Number of transcripts per chromosome.

We applied rigorous filters to identify potentially new lncRNAs and to discard possible protein-coding transcripts, as shown in Figure 1. For the merging of all samples’ GTF files, StringTie tool was used to obtain a complete GTF file with 991,412 transcripts. This file was used for further analysis, such as the removal of transcripts with coding potential, as well as those presenting a single exon and smaller than 200 nt. Subsequently, the tools for predicting coding and non-coding ability were used, reaching 871 transcripts in common to all tools (Figure 2a, Supplementary Table S2). From those, 699 were intronic lncRNAs (i), 170 intergenic (u), two antisenses (x), and one was a potentially new isomorph (j). The intronic transcripts showed the highest proportion (80%) of lncRNA candidates identified (Figure 2b). The lncRNAs found had a smaller exon number than the protein-coding genes and approximately 93% of the lncRNAs had only two exons (Figure 2c). The lncRNAs ranged from 221 to 60,446 bp in length, with an average of 5840 bp (Figure 2d), and were located in all swine chromosomes (Figure 2e). The results of the four codification prediction tools are presented in Supplementary Tables S3–S6.

### 3.3. Differentially Expressed lncRNAs

**Figure 3:**
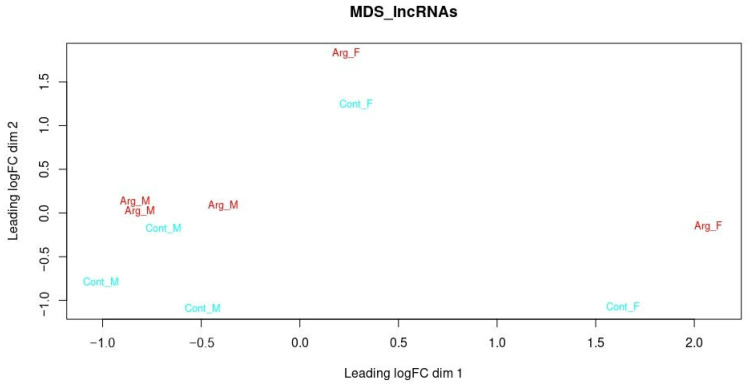
Multidimensional scaling of the samples. F, Female; M, Male; CONT, without supplementation; and ARG, with arginine supplementation.

First, to visualize the similarity structure inherent to the dataset, the Multidimensional Scaling (MDS) plot was a useful tool for identifying our grouping patterns (Figure 3). We visualized the greater separation of the samples concerning sex. There was no interaction (*p* > 0.05) between the factors studied (Supplementary Table S7). Therefore, the factors were evaluated separately. No differences were found between ARG and CONT fetuses regarding the identification of lncRNAs (Supplementary Table S8).

For the sex factor, seven lncRNAs, ENSSSCG00000053329, ENSSSCG00000049587, ENSSSCG00000044223, ENSSSCG00000044023, ENSSSCG00000057564, ENSSSCG00000043846, ENSSSCG00000055301, and a potentially new M STRG.22328.1, were differentially expressed between male and female pig fetuses at 35 days of age (Table 2). The lncRNAs were all upregulated in males.

### 3.4. Neighboring Genes of the DE lncRNAs

The lncRNAs do not encode proteins, which represents a major challenge in the identification of their main function. In this study, we investigated the function of lncRNAs by analyzing the protein-coding genes close to 300 kb up and downstream from the DE lncRNAs, naming this as cis-regulatory function.

For the effect of sex, we found 33 genes co-located together to the already described DE lncRNAs, 16 of which were located upstream and 17 downstream (Table 3). Subsequently, we performed GO and KEGG pathway enrichment analyses of co-localized mRNAs to predict the functions of differentially expressed lncRNAs. However, no significant GO enrichment results were obtained, since the number of co-localized mRNAs was low.

### 3.5. Potentially New DE lncRNAs Concerning Sex

From the 871 potentially new lncRNAs found, one was DE, MSTRG.22328.1, and was located on the Y chromosome (10380524-10381244) at the reverse strand, having three exons and 855 nt, thus being classified as an intergenic lncRNA. MSTRG.22328.1 is located downstream to the coding genes AMELY (Amelogenin Y-Linked) and ENSSSCG00000039365 and upstream to LOC110255257 (oral–facial–digital syndrome 1 protein-like) and ENSSSCG00000063414. As no information about this potentially new lncRNA was found, no further inferences about it will be made. More information on its sequence can be found in Supplementary File S2.

## 4. Discussion

In mammals, sex is determined by a pair of sexually dimorphic chromosomes (X and Y), with males being the heterogametic sex (XY) and females the homogametic (XX). The X and Y sex chromosomes contain 1412 and 131 genes in pigs, respectively, of which 37.89% and 31.30% are non-coding, with the majority being lncRNAs. The X chromosome contains 377 lncRNAs, while the Y chromosome contains 36 lncRNAs. LncRNAs play an important role in the mechanism of epigenetic regulation, impacting sex determination and differentiation in humans and animals [40,41].

LncRNAs can regulate the expression of genes located at cis chromosomes [42]. They can act as activators or repressors of transcription, indirectly influencing the expression of associated genes. Additionally, some lncRNAs are involved in the three-dimensional organization of chromosomes, which can affect the accessibility of genes to transcription factors. This spatial organization is important for gene expression regulation [43,44].

The lncRNA X-inactive specific transcript (lncRNA XIST) is necessary for the initiation of X chromosome inactivation [45,46], along with the lncRNAs TSIX and XACT, which also function in the regulation of X chromosome inactivation [47,48]. However, few studies have considered the possibility that the Y chromosome contains genes encoding regulatory RNAs. Our study has shown six differentially expressed lncRNAs on the Y chromosome and a potential novel one, indicating that they probably play important roles in spermatogenesis and sex determination, although most of their function is still unknown.

The lncRNA-mediated modulation of epigenetic modification is known to affect several biologically critical genes that can alter chromatin conformation by binding directly to target genes [49]. The lncRNA ENSSSCG000000053329 is downstream of the *DDX3Y* (DEAD-box helicase 3 Y-linked) gene and can regulate its performance in germ cell development, cell differentiation, and especially in spermatogenesis, particularly in promoting meiotic division [50,51,52,53].

The ENSSSCG00000049587 is upstream to *KDM5D* (Lysine Demethylase 5D), which is a histone demethylase located at the Y chromosome that can also regulate gene expression [54]. *KDM5D* is highly upregulated in the differentiation of male adult human neural crest-derived stem cells (NCSCs), being a critical factor in the osteogenic differentiation observed in male craniofacial NCSCs [55], in addition to participating in the regulation of gene expression through the demethylation of H3K4me1 and/or H3K4me3 in male mouse embryonic fibroblasts [56]. That is, lncRNAs can repress the expression of this gene at the transcriptional level and these epigenetic modifications play a fundamental role in regulating gene expression.

The lncRNAs ENSSSCG00000044223 and ENSSSCG00000044023 are located close to the genes *AMELY* (Amelogenin Y-Linked), *ZFY* (Zinc Finger Protein Y-Linked), and *EIF1AY* (Eukaryotic Translation Initiation Factor 1A Y-Linked), which are situated at the Y chromosome and play specific roles in sex determination, the development of secondary sexual characteristics, and functions related to the male sex [57,58,59].

The lncRNAs ENSSSCG00000057564 and ENSSSCG00000043846 can function as activators of gene expression by interacting with the promoter regions of nearby genes ENSSSCG00000025253 and *DDX3Y* (DEAD-box helicase 3 Y-linked), or by engaging with transcription factors that regulate their expression [60]. This interaction may facilitate transcription, subsequently increasing messenger RNA (mRNA) levels. Aside from their role in transcriptional regulation, lncRNAs can also impact the stability and translation of mRNA derived from neighboring genes, thereby influencing the quantity of produced proteins [61]. The depletion or malfunction of the *DDX3Y* gene has been associated with a pre-meiotic interruption in spermatogenesis [53]. The interplay between these lncRNAs and their nearby genes, primarily located on the Y chromosome, constitutes a burgeoning area of research, with many intricacies yet to be fully elucidated.

Although research on the function of Y chromosome lncRNAs is still evolving, it is clear that they play a complex role in regulating sexual biology. Understanding how lncRNAs influence gene expression and Y chromosome function is fundamental for understanding sex determination, as well as investigating sexual development.

Furthermore, not only the sex chromosomes are involved in sex differentiation or development, but many genes and transcripts in the autosomal chromosomes may also be involved. For example, we have found that the lncRNA ENSSSCG00000055301, located at chromosome 12, was upregulated in males, in which the genes *COPS3* (COP9 Signalosome Subunit 3), *UP22* (Ubiquitin-Specific Peptidase 22), *FLCN* (Folliculin), *PLD6* (Phospholipase D Family Member 6), and *MPRIP* (Myosin Phosphatase Rho-Interacting Protein) are upstream to its sequence.

The *COPS3* subunit of the COP9-signaling body [62] plays a crucial role in the deubiquitination and the regulation of protein kinase activity in several cellular processes [63]. The deubiquitinating enzyme *USP22* can remove ubiquitin from both histones H2A and H2B in the cell nucleus. *USP22* regulates transcription, modulating the levels of histone ubiquitination, thus influencing gene expression [64], and it is of great importance for the progression of the cell cycle, as its depletion results in an arrest of the cycle cell in the G1 phase [65].

*FLCN* is the gene responsible for encoding the Folliculin protein. This protein acts as a specific GTPase activator (GAP) for small GTPases, such as Rag GTPases. The activity of *FLCN* as a GAP in Rag GTPases is essential to recruit the mTORC1 complex (mTOR target of rapamycin complex) and the transcriptional factors TFEB and TFE3 to the lysosome [66]. TFEB/TFE3 are central controllers of lysosome biogenesis and functioning, playing an essential role in the regulation of autophagy. *FLCN* also acts as a regulator of guanine nucleotide exchange for the Rab35 protein. This action plays an important role in modulating the intracellular trafficking of the epidermal growth factor receptor [67].

The *PLD6* gene plays a pivotal role in generating primary RNAs that interact with piwi (piRNA), thereby regulating both male germ cell development and genome stability [68]. Male mice with a low PLD6 expression exhibit a significant decrease in piRNA quantity during spermatogenesis, leading to the blockade of meiosis [69].

The *MPRIP* gene encodes a cytoskeletal protein involved in the regulation of stress fibers [70]. Its depletion promotes an increase in the number of stress fibers in smooth muscle cells through the stabilization of actin fibers by phosphorylated myosin, and overexpression leads to the breakdown of stress fibers in neuronal cells [71,72].

For the lncRNA ENSSSCG00000055301’s downstream genes *DHRS7B* (Dehydrogenase/Reductase SDR Family Member 7B), *TMEM11* (Transmembrane Protein 11), *NATD1* (N-Acetyltransferase Domain-Containing 1), and *MAP2K3* (Mitogen-Activated Protein Kinase Kinase 3), their male-specific functions are not yet well understood, but they may be related to cell signaling processes, metabolism, or the regulation of specific metabolic pathways.

## 5. Conclusions

A total of 871 potentially new lncRNAs were characterized in the current study. Moreover, eight lncRNAs were found to be differentially expressed for sex in pig fetuses at 35 days after gestation. The potentially novel lncRNAs identified in our study provide a basis for future studies on the biological functions of lncRNAs in pigs. On top of that, in the near future, with the pig genome being more completely annotated, the description of lncRNA targets will be more clearly understood. The regulatory functions of lncRNAs are still being understood. Its diversity, low level of expression, and sequence divergence are challenges to be elucidated to understand their action. Advances in computational technologies, combined with improvements in our prediction abilities, will be important to determine their functions and understand the mechanism of action of the lncRNAs characterized in the current study.

## Figures and Tables

**Table 1:** Sample mapping of lncRNA transcripts on the swine genome.

Sample	Mappead_Reads	Mapping (%)
8209_E1	25,445,184	85.26
8218_E1	27,694,122	83.57
8218_E5	27,209,218	83.57
8219_E1	32,010,420	83.95
8219_E2	27,031,814	84.01
8219_E5	23,178,930	82.75
8273_E1	23,651,782	84.14
8274_E1	22,753,549	84.46
8274_E5	24,454,713	83.09
8284_E5	20,739,162	83.28

**Table 2:** lncRNAs differentially expressed concerning sex in swine fetuses at 35 days of age.

Ensembl ID	logFC ^1^	adj.P.Val ^2^	Strand
ENSSSCG00000053329	6.737	7.14 × 10^−6^	Forward
ENSSSCG00000049587	6.575	1.28 × 10^−5^	Forward
ENSSSCG00000044223	5.490	5.78 × 10^−5^	Reverse
ENSSSCG00000044023	5.327	0.000242	Forward
ENSSSCG00000057564	5.117	0.000242	Forward
ENSSSCG00000043846	4.985	0.00036	Reverse
ENSSSCG00000055301	4.347	0.006892	Forward
MSTRG.22328.1	5.938	2.12 × 10^−5^	Reverse

^1^ Log2-fold change values through contrasts between 35-day-old female and male fetuses; ^2^ Adjusted *p*-value for multiple correction tests to reduce type I error; significance threshold: adjusted *p*-value < 0.05 and |logFC| > 0.5.

**Table 3:** LncRNAs differentially expressed between male and female pig fetuses at 35 days of gestation and their neighboring upstream and downstream genes.

lncRNAs	Gene Upstream	Gene Downstream
*ENSSSCG00000053329*	*LOC100626157*, *ENSSSCG00000025253*	*DDX3Y*
*ENSSSCG00000049587*	*KDM5D*	*LOC110257970*, *LOC110257894*
*ENSSSCG00000044223*	*LOC100624149*, *ENSSSCG00000052941*, *EIF1AY*, *LOC102162178*	*ZFY*, *AMELY*
*ENSSSCG00000044023*	*ZFY*, *LOC100624149*, *ENSSSCG00000052941*, *EIF1AY*	*AMELY*, *ENSSSCG00000039365*
*ENSSSCG00000057564*	*ENSSSCG00000025253*	*ENSSSCG00000026430*, *ENSSSCG00000027046*
*ENSSSCG00000043846*	*ENSSSCG00000025253*	*DDX3Y*, *LOC100625207*
*ENSSSCG00000055301*	*COPS3*, *FLCN*, *PLD6*, *MPRIP*, *ENSSSCG00000040134* and *USP22*	*DHRS7B*, *TMEM11*, *NATD1*, *MAP2K3*, *ENSSSCG00000051348* and *ENSSSCG00000034773*

## Data Availability

Data are available upon request.

## Supplementary Materials

The following are available at: https://www.mdpi.com/article/10.3390/ani14131897/s1. Animal_Supplementary Materials. Table S1: Transcript StringTie. Table S2: Potentially new LncRNAs. Table S3: CNCI program’s non-coding transcripts. Table S4: CPC2 program’s non-coding transcripts. Table S5: PLEK program’s non-coding transcripts. Table S6: CPAT program’s non-coding transcripts. Table S7: Interaction between sex and treatment (CONT-ARG). Table S8: Treatment control and arginine. File S1: Script_lncRNAs_DE. File S2: 871_lncRNAs.
