# Supplementary material for: Long Non-Coding RNAs Differentially Expressed in Swine Fetuses
Source: Animals (Basel). 2024 Jun 27;14(13):1897. doi: 10.3390/ani14131897 (PMC11240794; doi:10.3390/ani14131897)
Supplement: Supplementary file 1 [file animals-14-01897-s001.zip › animals-2977992-File S2.pdf]

```
###Analysis of differentially expressed lncRNAs
##Pig fetuses
###Francelly Campos
##==============================================================================
===============================================
##Load the packages
##If you don't have them, install them
library ("limma")
library("gplots")
library("ggplot2")
library("edgeR")
library(readxl)
library(plotly)
library("data.table")

#Call the counts
setwd("/localData/alunos/francelly.campos/LncRNAs/DE_lncRNAs/")

countData <- as.matrix(read.csv("871_transcritos_counts.csv",
row.names="transcript_id"))

##Create a txt file with your experiment information
amostras <- read.table(file = "amostra.txt", header=T)
class(amostras)

##Delete column X countData (If you leave column X = error)
count <- countData[,-1]

dge <- DGEList(counts = count, samples = amostras,
               group = NULL, genes = NULL, remove.zeros = FALSE)
class(dge)
dim(dge$counts)
d.full <- dge # keep the old one in case we mess up
##==============================================================================
=============================================
##Creating new objects that will be included in the DGE-list later

samplenames <- amostras$SAMPLE_ID
samplenames

sex <- as.factor(amostras$Sex)
sex

group <- as.factor(amostras$Treatment)
group

mae <- as.factor(amostras$mae)
mae

##Data pre-processing

cpm <- cpm(dge)
lcpm <- cpm(dge, log=TRUE)
```

```
#write.csv(cpm, file = "tabela_cpm.csv")

##Transformations from the raw-scale

L <- mean(dge$samples$lib.size) * 1e-6
M <- median(dge$samples$lib.size) * 1e-6
c(L, M)

L_million <- mean(dge$samples$lib.size)
M_million <- median(dge$samples$lib.size)
c(L_million, M_million)

##===============================================================================
=============================================
##Removing genes lowly expressed

table(rowSums(dge$counts==0)==10)

keep.exprs <- filterByExpr(dge, group=group)
dge <- dge[keep.exprs,, keep.lib.sizes=FALSE]
summary(keep.exprs)
##===============================================================================
==============================================
#To build the figures with raw and filtered data:

lcpm.cutoff <- log2(10/M + 2/L)
library(RColorBrewer)
#pdf('Raw_filtered_LMD_final.pdf')
nsamples <- ncol(dge)
col <- brewer.pal(nsamples, "Paired")
par(mfrow=c(1,2))
plot(density(lcpm[,1]), col=col[1], lwd=2, ylim=c(0,0.40), las=2, main="",
xlab="")
title(main = "A. Raw data", xlab = "Log-cpm")
abline(v=lcpm.cutoff, lty=3)
for (i in 2:nsamples){
  den <- density(lcpm[,i])
  lines(den$x, den$y, col=col[i], lwd=2)
}
#legend("topright", samplenames, text.col=col, bty="n")
lcpm <- cpm(dge, log=TRUE)
plot(density(lcpm[,1]), col=col[1], lwd=2, ylim=c(0,0.26), las=2, main="",
xlab="")
title(main="B. Filtered data", xlab="Log-cpm")
abline(v=lcpm.cutoff, lty=3)
for (i in 2:nsamples){
  den <- density(lcpm[,i])
  lines(den$x, den$y, col=col[i], lwd=2)
}

x2 <- dge
x2$samples$norm.factors <- 1
x2$counts[,1] <- ceiling(x2$counts[,1]*0.05)
x2$counts[,2] <- x2$counts[,2]*5
```

```
par(mfrow=c(1,2))
lcpm <- cpm(x2, log=TRUE)
boxplot(lcpm, las=2, col=col, main="")
title(main="A. Example: Unnormalised data",ylab="Log-cpm")
x2 <- calcNormFactors(x2)
x2$samples$norm.factors

lcpm <- cpm(x2, log=TRUE)
boxplot(lcpm, las=2, col=col, main="")
title(main="B. Example: Normalised data",ylab="Log-cpm")
##================================================================================
============================================
## Normalising gene expression distributions

dge <- calcNormFactors(dge)
logCPM <- cpm(dge, log=TRUE, prior.count=2)
##================================================================================
=============================================
### MDS and PCA plot**
par(mfrow=c(1,1))

sample_id = apply(amostras[,c("SAMPLE_ID","Sex","Treatment")],1,function(x)
paste(na.exclude(x),collapse="_"))

plotMDS(dge, label=sample_id, col =ifelse(group=="group", "darkviolet",
"deeppink") ,cex=0.8,main="PCA", gene.selection = "common")


plotMDS(dge, label=sample_id, col =
rainbow(length(levels(factor(group))))[factor(group)],cex=0.9,main="MDS ")
##================================================================================
=============================================
##Design
###Random effect correlation:

design <- model.matrix(~Sex + Treatment + Sex*Treatment, data=dge$samples)
dupC <- duplicateCorrelation(logCPM, design, block=dge$samples$mae)
dupC$consensus
##================================================================================
===============================================
###Differential expression analysis

fit <- lmFit(logCPM, design, block=dge$mae, correlation=dupC$consensus)
fit <- eBayes(fit, trend=TRUE, robust=TRUE)

interacion <- topTable(fit, coef=4, number = nrow(dge))
write.csv(interaçao, "interation_lncRNAs_35d.csv", quote = FALSE, row.names =
TRUE)

Trat <- topTable(fit, coef=3, number = nrow(dge)) #trat effect
write.csv(Trat, "Trat_lncRNAs_35d.csv", quote = FALSE, row.names = TRUE)

Sex <- topTable(fit, coef=2, number = nrow(dge)) #sex effect
```

```
write.csv(Sex, "Sex_lncRNA_35d.csv", quote = FALSE, row.names = TRUE)
```
