# Supplementary material for: Long Non-Coding RNAs Differentially Expressed in Swine Fetuses
Source: Animals (Basel). 2024 Jun 27;14(13):1897. doi: 10.3390/ani14131897 (PMC11240794; doi:10.3390/ani14131897)
Supplement: Supplementary file 1 [file animals-14-01897-s001.zip › File S1. Sequence of potential new lncRNAs (1).pdf]

```
>MSTRG.22328.1 gene=MSTRG.22328 855
GCTGTGGAATCTCGCGAGGGTAGCTTCGGGAGTCCCCACTGCAGCTGCGGGGCGCGGGTCTCTG
CCGCGGGGACTGACTGGGCGTTTCCGTTGCCGGCGTCTCGCCGCAGCAGAGGAATGCTTCCTGG
TATCAGCACTGCTGTGGCTCTGAGGGTACACAGACTGTCATCCAGGCTTCAGGTCTCCAGGGTG
TTCTAAGAAACTCTCGAATTCCTTGGCTCACTGCCTCTTCCTCCACCTTCAAAGCCAGCATCAA
GGTACCTCCCTGCACCACCTTCTGCAACTCCACCCTGCGCCTGGATGAGCCTGCCCTCTTTCCC
TTTAAAGAACCTGGTAATTACAGGGAGCCCAGGTGAGTAGTCCAGGCTCCTCCCCGCCCAAGGG
GCTGCTGCTGAGGGGCCTGAGCCCTTTGTCCGGTAAAGCAGCCCGCACAGGTTCCAGGGCTCAG
AGTGTGGGCATCTTTGGGAGGGGCAGCTGTTCTGGCTCCTCCACATTTTCAAAATCAGCCTGTC
CCTTTTCGTCCCTCTTCCTTCCCTTTCCTTCTTCCTTTCTCGGTGAAGAGCAGCGTTATCTTGC
TATTCCTCTGCATCACCTCAGCCAGACATGTGGAAACATCCTCGGTTCTAGCCTCTTCGAAGCC
TCAGTTCCCGGTTAGGCCCGTGCCAGGTCTGCAGCCACCCCCACACCTCTGGAGATGCTGGGAG
GGCCCCTGTTTCAGCGAGGGGACGGGTGAGTCGGGGAGAGCGCCTCCCTCCTCCCAGACAGCAC
GCAGCCCAGTGTGGGACTCACCTCTGCGCCCGCCCCCGCCCAGTAAGCCCCAGTCCCTTCCTGA
GACGCCGCCTTCCTTTACCCCCA
```
